# Differential effect of HLA class-I *versus* class-II transgenes on human T and B cell reconstitution and function in NRG mice

**DOI:** 10.1038/srep28093

**Published:** 2016-06-21

**Authors:** Sai Majji, Wathsala Wijayalath, Soumya Shashikumar, Luis Pow-Sang, Eileen Villasante, Teodor D. Brumeanu, Sofia Casares

**Affiliations:** 1 US Military Malaria Vaccine Program, Naval Medical Research Center/Walter Reed Army Institute of Research, Silver Spring, MD, USA; 2 Department of Medicine, Uniformed Services University of Health Sciences, Bethesda, MD, USA.

## Abstract

Humanized mice expressing Human Leukocyte Antigen (HLA) class I or II transgenes have been generated, but the role of class I *vs* class II on human T and B cell reconstitution and function has not been investigated in detail. Herein we show that NRG (NOD.RagKO.IL2RγcKO) mice expressing HLA-DR4 molecules (DRAG mice) and those co-expressing HLA-DR4 and HLA-A2 molecules (DRAGA mice) did not differ in their ability to develop human T and B cells, to reconstitute cytokine-secreting CD4 T and CD8 T cells, or to undergo immunoglobulin class switching. In contrast, NRG mice expressing only HLA-A2 molecules (A2 mice) reconstituted lower numbers of CD4 T cells but similar numbers of CD8 T cells. The T cells from A2 mice were deficient at secreting cytokines, and their B cells could not undergo immunoglobulin class switching. The inability of A2 mice to undergo immunoglobulin class switching is due to deficient CD4 helper T cell function. Upon immunization, the frequency and cytotoxicity of antigen-specific CD8 T cells in DRAGA mice was significantly higher than in A2 mice. The results indicated a multifactorial effect of the HLA-DR4 transgene on development and function of human CD4 T cells, antigen-specific human CD8 T cells, and immunoglobulin class switching.

Humanized mice able to engraft human hematopoietic stem cells (HSC) and to reconstitute a human immune system can be used to investigate the development of human immune cells. They may also represent new pre-clinical models to evaluate the therapeutic efficacy of human vaccine candidates prior to clinical trials^1^,^2^. A major landmark for generation of humanized mouse models was the inclusion of the murine IL-2 receptor gamma chain KO (IL2Rγc) mutation in immunodeficient (RAG or *scid*) NOD mice that proved to abolish mouse innate natural killer (NK) activity and improve human HSC engraftment^3^,^4^. However, the humanized IL2RγcKO mice still showed poor human T cell reconstitution and function^5^, which was attributed to the lack of HLA expression as a critical element to support homing and education of human pro-T cells in the mouse thymus. To test this hypothesis, we and others have generated transgenic (Tg) humanized mice expressing HLA class II (hybrid human/mouse DR0401, DR0405, or DR0101) molecules under the mouse I-E promoter, or HLA class I (human A2.01, human B51.01, or human/mouse A2.01) molecules under the HLA class I promoter (Table 1). The HLA-Tg humanized mice were infused with HLA-matched human hematopoietic stem cells (HSC) and assessed for efficacy of reconstituting human T and B cells^6^,^7^,^8^,^9^,^10^,^11^,^12^,^13^,^14^,^15^. The studies showed improved human T cell function in the HLA-Tg humanized mice but their ability to reconstitute higher numbers of human T cells as compared to control (non-Tg) mice or to undergo immunoglobulin class switching has not come to a consensus^6^,^7^,^8^,^9^,^10^,^11^,^12^,^13^,^14^,^15^. In addition, one study showed lower human T cell reconstitution in HLA-A2 Tg mice as compared to non-Tg mice^9^. Such contrasting results may be explained by differences in the methodology used by different laboratories using humanized mice that refer to (i) the critical mutations needed to abolish the mouse adaptive immune system (*scid* mutation in NSG and NOK mice, or RAGKO mutation in NRG mice) and mutations to decrease mouse innate activity (IL2RgcKO in NSG and NRG mice or Jak3KO in NOK mice) (ii) the structure of the HLA transgenes (human or hybrid human/mouse), (iii) the timing of HSC infusion (neonatal or adult mice), the conditioning radiation dose (100 to 350 rads), and route for HSC infusion (intravenous or intrahepatic) (iv) the source of HSCs (umbilical cord blood, fetal liver, or adult bone marrow), (v) HSC preparations infused (CD34^+^ enriched or T-cell depleted), and (vi) the numbers of HSC infused per mouse (5 × 10^3^ to 5 × 10^5^) (reviewed in Table 1)^6^,^7^,^8^,^9^,^10^,^11^,^12^,^13^,^14^,^15^.

To compare the effect of transgenic HLA class I *vs* class II expression on human T-cell reconstitution and function as well as on human B cell immunoglobulin class switching, we used three humanized mouse strains in the NRG (NOD.RagKO.IL2RgcKO) background expressing either HLA-A2.1 molecules (hereafter referred as to A2 mice), or HLA-DR4 molecules (DRAG mice), or co-expressing HLA-A2.1 and HLA-DR4 molecules (DRAGA mice). The HLA-A2.1 transgene encodes for a hybrid human/mouse α chain (HLA-A2.1α1α2/H-2D^b^) covalently linked to human β2-microglobulin^16^, and this transgene has been tested by several laboratories in the NSG background (NOD.*scid*.IL2R*g*cKO)^6^,^7^,^8^. The HLA-DR4 molecule is encoded by hybrid human/mouse α (HLA-DR0101α1/IE^d^α) and β (HLA-DR0401β1/IE^d^β) transgenes, and it has been previously tested in the NRG background (DRAG mice)^12^. The DRAGA, DRAG, and A2 mice were infused in the adulthood (4 to 6 week-old) by the intravenous route with negatively isolated human CD34^+^ HSC from umbilical cord blood. Herein, we show that the transgenic expression of HLA-DR4 molecules in DRAG and DRAGA mice significantly improves human T-cell reconstitution, CD4 T and CD8 T cell function, and B-cell immunoglobulin class switching as compared to transgenic expression of HLA-A2 molecules alone. Secondly, co-expression of HLA-DR4 and HLA-A2 molecules in DRAGA mice significantly increases the numbers and cytotoxic activity of antigen-specific human CD8 T cells as compared to A2 mice.

## Results

### HLA-DR4 (class II), but not HLA-A2 (class I) expression in NRG mice improves human T cell reconstitution rates

**Figure 1:**
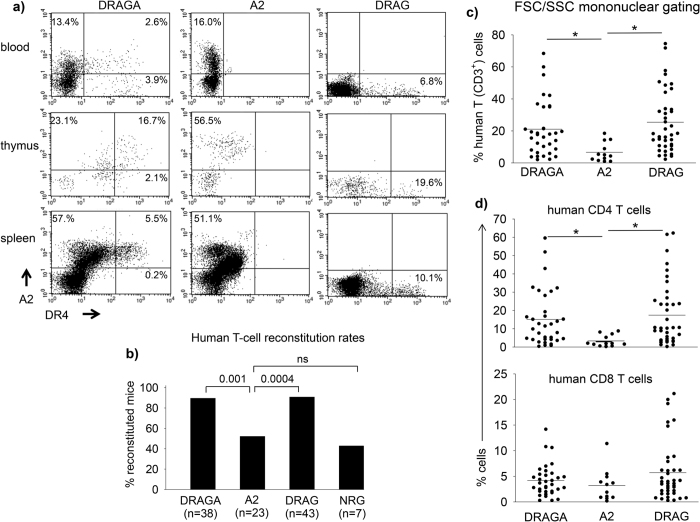
Human T-cell reconstitution in peripheral blood of humanized HLA-Tg mice. Panel (**a**) FACS analysis of blood, thymus and spleen of naïve (non-HSC infused) DRAGA, A2, and DRAG mice stained with HLA-A2 and HLA-DR4 Abs. Panel (**b**) four-to-six week old mice were infused with HLA-A2/DR4-positive HSC (10^5^/mouse, Supplementary Table S1) and examined 16–18 weeks later for reconstitution of human T cells in peripheral blood by FACS using CD3, CD4, and CD8 Abs. Data represent the percentage of mice having human T cells in blood. The cut-off for positive human CD3^+^ T cells was calculated as three times the standard deviation over the background levels of cells from naıve (non-HSC infused) DRAG mice that were stained with anti-human CD3 (0.17%). Z test indicated that the human T cell reconstitution rate in A2 mice (12 of 23) and NRG (3 of 7) was similar (p = 0.66), but significantly lower as compared to DRAGA (34 of 38, p = 0.001) and DRAG (39 of 43, p = 0.0004) mice. Panels (**c,d**) frequency of human T cells (CD3^+^), and human CD4 T and CD8 T cell subsets, in the reconstituted DRAGA, A2 and DRAG mice. Data represent values in individual mice on a mononuclear FSC/SSC gating. Horizontal lines represent mean values. Student *t* test indicated that A2 mice reconstituted significantly lower frequencies of total human T cells and CD4 T cells than DRAGA and DRAG mice (*p < 0.05), but similar frequencies of human CD8 T cells.

We previously showed that humanized NRG mice expressing HLA-DR4 molecules (DRAG mice) have a significantly higher human T-cell reconstitution rate (percentage of mice able to develop human T cells), and develop significantly higher numbers of human CD4 T cells than the control NRG mice^12^. To address the role of HLA class I *vs* class II molecules on human T cell reconstitution and function, we generated transgenic NRG mice co-expressing HLA-A2 and HLA-DR4 molecules (DRAGA mice) or expressing only HLA-A2 molecules (A2 mice). Figure 1a shows that DRAGA mice co-express HLA-A2 and HLA-DR4 molecules, while A2 mice express only HLA-A2 molecules. As we previously reported^12^, the DRAG mice express only HLA-DR4 molecules (Fig. 1a). DRAGA, DRAG, A2, and control non-transgenic (Tg) NRG mice were injected intravenously with HLA-A2.1/DR0401 human HSC from the same donors (Supplementary Table S1), and 16–18 weeks later, mice were examined for human T cell reconstitution in the peripheral blood by FACS using human CD3 antibodies. As illustrated in Fig. 1b, the DRAGA and DRAG mice showed a similar human T-cell reconstitution rate (34 of 38 DRAGA mice and 39 of 43 DRAG mice), which was significantly higher than in the A2 mice (12 of 23 mice) and in control non-Tg NRG mice (3 of 7 mice). Of note, the rate of human T cell reconstitution in DRAG and non-Tg NRG mice as found in this study was similar to that reported in our previous study^12^. These results indicated that the expression of HLA-DR4, but not HLA-A2, molecules significantly increases the ability of NRG mice to reconstitute human T cells.

### HLA-DR4, but not HLA-A2, expression in NRG mice increases the numbers of human CD4 T cells, but neither HLA-DR4 nor HLA-A2 increases the numbers of human CD8 T cells

We next compared the frequency of human T cells in the blood of DRAGA, DRAG, and A2 mice by FACS using human CD3 Abs. Of note, mice that were not able to reconstitute human T cells in blood were excluded to allow strict comparison on human T cell numbers on the reconstituted mice. Also, the T cell frequencies presented correspond to mononuclear FSC/SSC gating. As shown in Fig. 1c, the frequency of human T cells (CD3^+^) in the blood of DRAGA and DRAG mice was similar, and significantly higher than in the A2 mice.

The human CD3^+^ T cell compartment is comprised of two major cell subsets based on the surface expression of CD4 or CD8 co-receptors. Human CD4^+^ T cells recognize peptides in the context of HLA-class II molecules while CD8^+^ T cells recognize peptides in the context of HLA-class I molecules^17^. Thus, we analyzed the frequency of human CD4 and CD8 T cell subsets in the peripheral blood of T cell reconstituted DRAGA, DRAG, and A2 mice. Figure 1d shows that DRAGA and DRAG mice had similar frequencies of human CD4 T cells which were significantly higher than those in the A2 mice. However, the frequency of human CD8 T cells in peripheral blood was comparable among the DRAGA, DRAG, and A2 mice (Fig. 1d, lower panel).

**Figure 2:**
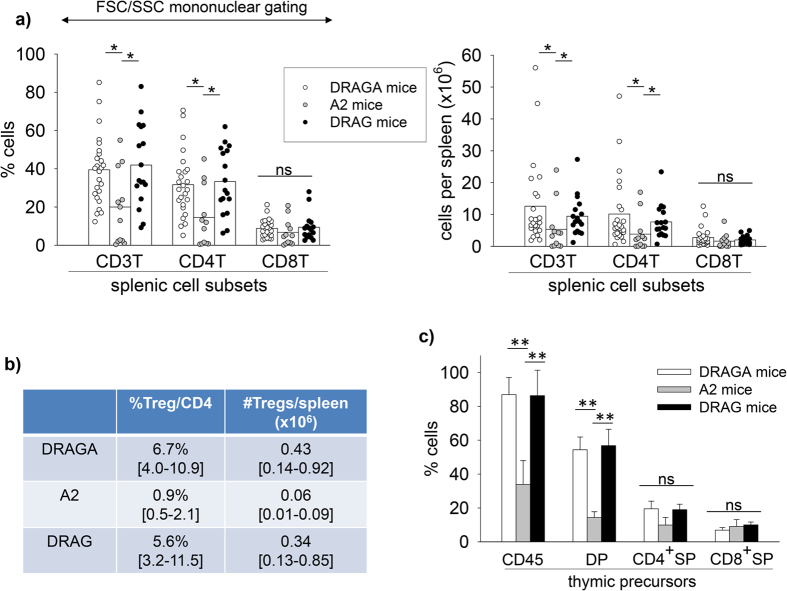
Human T cell reconstitution in lymphoid organs of humanized HLA-Tg mice. At 16–18 weeks post-infusion of HSCs, DRAGA, DRAG, and A2 mice were examined for the frequency of human CD4 T and CD8 T cells in spleen and thymus by FACS using human CD3, CD4, and CD8 Abs. Panel (**a**) frequency (left) and total numbers (right) of human T cells (CD3^+^) and human CD4 and CD8 T cell subsets in spleens of DRAGA (n = 25), DRAG (n = 17), and A2 (n = 12) mice. Data show values in individual mice on a mononuclear FSC/SSC gating. Bars indicate mean value for each group of mice. Student *t* test indicated that spleens of DRAGA and DRAG mice reconstituted significantly higher frequencies and cell numbers of human CD4 T cells than A2 mice (*p < 0.05). No significant differences were found for frequency and numbers of human CD8 T cells in the spleens of DRAGA, DRAG, and A2 mice (ns, not significant p > 0.05). Panel (**b**) shows the frequency and cell numbers of human CD3^+^ FOXP3^+^ T cells (Tregs) in the spleens of DRAGA (n = 8), DRAG (n = 6), and A2 (n = 4) mice. Student *t* test indicated that DRAGA and DRAG mice reconstituted higher numbers of human Tregs than A2 mice. Panel (**c**) Frequency of human cells (CD45^+^), double CD4^+^ CD8^+^ positive (DP), CD4^+^ CD8^−^ single positive (SP), and CD4^−^CD8^+^ SP cells in thymus of DRAGA (n = 9), DRAG (n = 11), and A2 (n = 7) mice. Data represent mean ± SD of mice analyzed individually. Student *t* test indicated that the thymus of DRAGA and DRAG mice reconstituted significantly higher frequencies of human CD45^+^ cells and DP cells than the A2 mice (**p < 0.005) but similar frequencies of SP cells (ns, not significant).

To determine whether the increased human CD4 T cell frequencies in the peripheral blood of DRAGA and DRAG mice resulted in higher numbers of human CD4 T cells repopulating the lymphoid organs, their spleens were examined by FACS. As illustrated in Fig. 2a, the DRAGA and DRAG mice had similar frequencies (left panel) and total numbers of human CD4 T cells per spleen (right panel), which were significantly higher than those in the spleens of A2 mice. These results clearly indicated that the expression of HLA-DR4 molecules but not HLA-A2 molecules, improves splenic repopulation with human CD4 T cells.

On the other hand, the frequency and numbers of human CD8 T cells in the spleens of DRAGA, DRAG, and A2 mice were comparable, indicating that neither HLA-A2 nor HLA-DR4 molecules increase the numbers of human CD8 T cells (Fig. 2a).

### HLA-DR4 (class II), but not HLA-A2 (class I) expression in NRG mice increases the numbers of human CD4^+^ FOXP3^+^ regulatory T cells (Tregs)

The regulatory CD4^+^ FOXP3^+^ cells (Tregs) represent an important CD4 T cell subset responsible for maintenance of self-tolerance^18^. We thus compared the ability of T cell reconstituted DRAGA, DRAG, and A2 mice to repopulate human Tregs in spleens. As illustrated in Fig. 2b, the spleens of DRAGA and DRAG mice contained similar frequencies and total numbers of human Tregs, which were significantly higher than those in A2 mice. The results indicated that expression of HLA-DR4 molecules enhances splenic reconstitution with human Tregs, whereas expressions of HLA-A2 molecules do not play a role.

### HLA-DR4 (class II), but not HLA-A2 (class I) expression in NRG mice favors thymic engraftment with human pro-T cells

Since T cells are developed in the thymus by engraftment of pro-T cells derived from bone marrow^19^, we examined the thymus of DRAGA, DRAG, and A2 mice showing reconstitution with human T cells in blood. Data in Fig. 2c show that the thymus of DRAGA and DRAG mice, as compared to A2 mice, were highly populated with human hematopoietic CD45^+^ cells and had higher frequencies of CD4^+^ CD8^+^ double positive (DP) cells. The mean frequency of CD4^+^ CD8^−^ single positive (SP) cells in the thymus of DRAGA and DRAG mice was higher than in A2 mice, though the results did not reach statistical significance (p = 0.07, Student *t* test). The frequency of CD4^-^CD8^+^ SP cells in the thymus of DRAGA, DRAG, and A2 mice were however quite similar (p = 0.4). These results indicated that in contrast to HLA-A2 expression, the expression of HLA-DR4 molecules in NRG mice significantly enhances thymic engraftment with human pro-T cells.

### HLA-DR4 (class II), but not HLA-A2 (class I) expression in NRG mice is required for development of functional human CD4 T and CD8 T cells able to secrete cytokines

**Figure 3:**
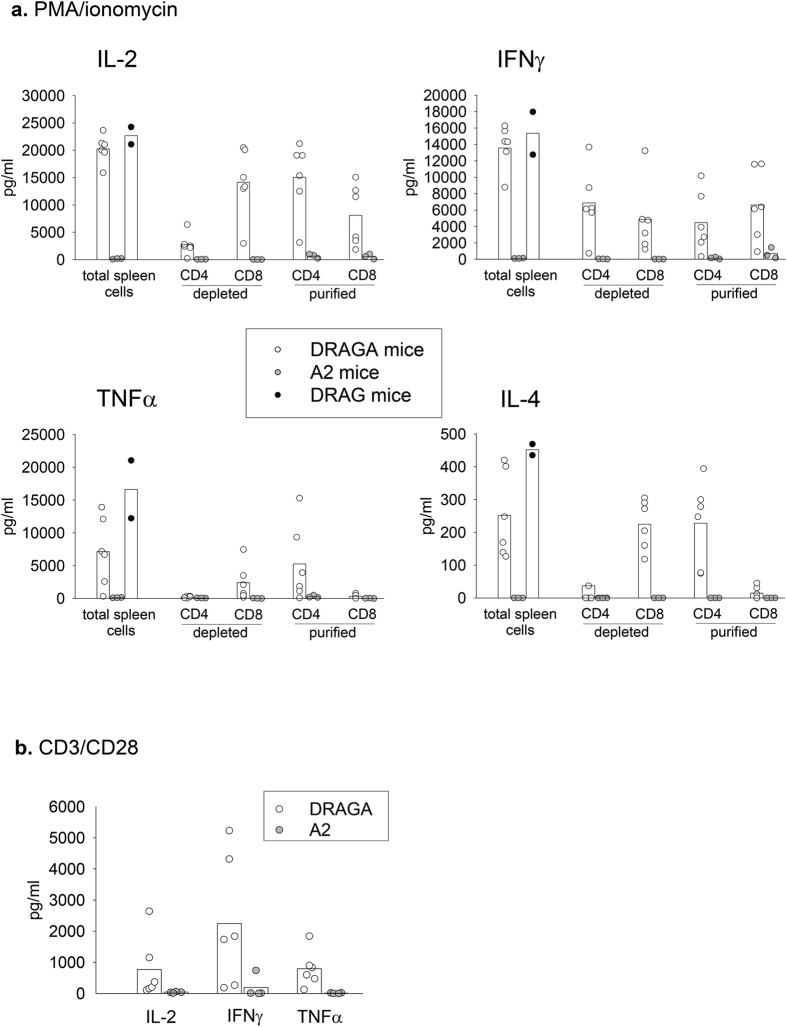
HLA-DR4 (class II), but not HLA-A2 (class I), expression in NRG mice is required for development of human CD4 T and CD8 T cells able to secrete cytokines. Panel (**a**) splenic cells from DRAGA (n = 6), A2 (n = 3) and DRAG (n = 2) mice were stimulated for 36 h with PMA/ionomycin, and the cytokines secreted in cell culture supernatants were measured by Luminex. Non-stimulated cells secreted < 50 pg/ml. Data present cytokine levels over the background (non-stimulated). To address the function of human CD4 and CD8 T cells from DRAGA and A2 mice, CD4 T cell-depleted or CD8 T cell-depleted spleen cells, or positively-isolated splenic CD4 T and CD8 T cells were stimulated as above. Data represent values of mice analyzed individually. Bars indicate average cytokine levels. Panel (**b**) splenic cells from additional groups of DRAGA (n = 6) and A2 (n = 4) mice were stimulated *in vitro* with anti-human CD3/CD28 coated beads and the levels of human IL-2, IFNγ, and TNFα secreted in cell culture supernantants were measured by luminex. Data represent values of mice analyzed individually. Bars indicate average cytokine levels.

One of the effector functions of human T cells is secretion of cytokines. Cytokines are small signaling molecules that regulate humoral and cellular responses^20^. We thus investigated the ability of human T cells from DRAGA, DRAG, and A2 mice to secrete human cytokines in cell culture supernatants upon *in vitro* stimulation with PMA and ionomycin. Luminex data in Fig. 3a show that the splenic human T cells from DRAGA mice secreted very high levels of human cytokines (IL-2:20,246 ± 2,555; IFNγ:13,748 ± 2,671; TNFα:7,131 ± 5,249; IL-4:250 ± 131 pg/ml), which were comparable to DRAG mice (22,667 ± 2,238; 15,362 ± 3,688; 16,631 ± 6,237; 452 ± 24 pg/ml). In contrast, the splenic T cells from A2 mice secreted very low levels of these cytokines (148 ± 75, 109 ± 17, 122 ± 23, and 0 pg/ml). The proficiency of human T cells from DRAG mice to secrete cytokines as found in this study is in agreement with our previous study^12^, which also demonstrated that both human CD4 T and CD8 T cell subsets from DRAG mice secreted high levels of human cytokines^12^. Thus, we assessed the function of CD4 T and CD8 T cells from DRAGA and A2 mice as we previously did for DRAG mice^12^. For this, splenic CD4-depleted or CD8-depleted cells, and purified CD4 T or CD8 T splenic cells were stimulated *in vitro* with PMA and ionomycin, and the cytokines secreted in cell culture supernatants were measured by Luminex. As shown in Fig. 3a, both CD4 and CD8 T cells from DRAGA mice strongly responded to stimulation, while CD4 T and CD8 T cells from A2 mice secreted very low levels of human cytokines.

Since PMA/ionomycin is a very strong T-cell stimulator that bypasses TCR signaling and directly activates cytokine gene transcription on T cells^21^,^22^,^23^, we also investigated the ability of human T cells from DRAGA and A2 mice to respond to TCR stimulation. For this, splenic T cells from additional groups of DRAGA and A2 mice were stimulated *in vitro* with anti-human CD3/CD28-coated beads and cytokines secreted in cell culture supernantants were measured by luminex. As illustrated in Fig. 3b, the T cells from DRAGA mice secreted high levels of human IL-2, IFNγ, and TNFα, while the T cells from A2 mice secreted low levels of these cytokines. In aggregate, these results clearly indicated that the transgenic expression of HLA-DR4 molecules suffices for reconstitution of functional human CD4 and CD8 T cells able to secrete cytokines, while the transgenic expression of HLA-A2 molecules alone does not.

### Co-expression of HLA-A2 and HLA-DR4 molecules in NRG mice increases the number and cytotoxic function of antigen-specific, HLA-A2-restricted human CD8 T cells

**Figure 4:**
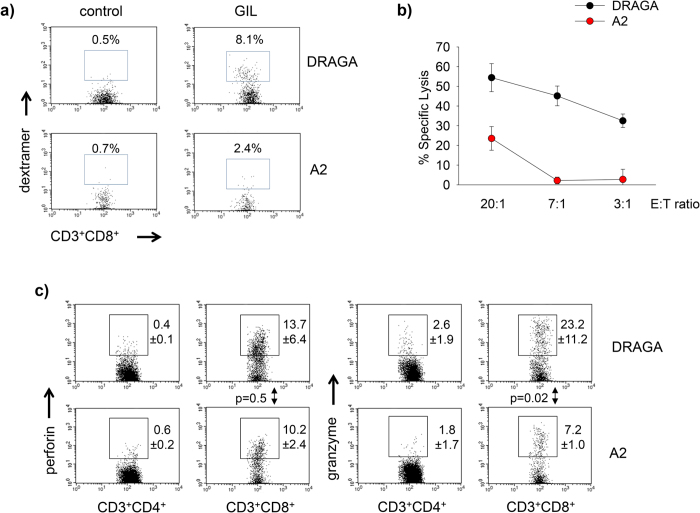
Co-expression of HLA-A2 and HLA-DR4 molecules in NRG mice increases the numbers and function of antigen-specific human CD8 T cells. Panel (**a**) DRAGA and A2 mice (n = 3 per group, infused with HSC from the same donor) were immunized intramuscularly twice at two weeks apart with GIL peptide (200 μg) encapsulated in liposomes (100 μl). Ten days after the last immunization, pooled splenocytes from the three mice were analyzed by FACS using human CD3, CD8, and HLA-A2/GIL dextramers. Control mice were non-immunized DRAGA and A2 mice (n = 2) that were infused with HSC from the same donor as the GIL-immunized mice. Data show the frequency of GIL-specific CD8 T cells in DRAGA and A2 mice. Panel (**b**) splenic cells from GIL-immunized DRAGA and A2 mice (n = 3 pooled) were stimulated *in vitro* with GIL peptide for seven days, and effector cells were incubated at various cell ratios with GIL-pulsed T2 target cells (effector:target, E:T) for 4 hours. Data points represent mean ± SD percent of specific lysis as measured by ^51^Cr release in triplicated samples. Panel (**c**), expression of intra-cellular perforin and granzyme B in splenic CD8 T and CD4 T cells from DRAGA (n = 7) and A2 (n = 3) mice. Data represent mean ± SD of mice analyzed individually.

Since both DRAGA and A2 mice express HLA-A2 molecules, we next compared their ability to reconstitute HLA-A2-restricted CD8 T cells. For this, groups of DRAGA and A2 mice (n = 3) infused with HSC from the same donor, were immunized twice at two weeks intervals with GIL peptide encapsulated in liposomes. Ten days after the last immunization, their spleens were analyzed for the frequency of splenic GIL-specific CD8 T cells by *ex vivo* dextramer staining. The GIL peptide is derived from the influenza A virus matrix protein and it is recognized by human CD8 T cells in the context of HLA-A2.1 molecules^24^. Controls were non immunized mice infused with HSC from the same donor as the GIL-immunized mice. Figure 4a shows that both DRAGA and A2 mice were able to elicit GIL-specific CD8 T cells, though the immunized DRAGA mice elicited almost 4 times more GIL-specific CD8 T cells (8.1%) than the immunized A2 mice (2.4%).

Since the main function of human CD8 T cells is to kill target cells expressing foreign peptides in the context of HLA class I molecules, we also compared the cytotoxic function of GIL-specific human CD8 T cells from the immunized DRAGA and A2 mice. For this, their splenic cells were stimulated *in vitro* with GIL peptide and IL-2 for 7 days, and then tested for cytotoxicity using T2 (HLA-A2.1) target cells pulsed with GIL peptide. As illustrated in Fig. 4b, the human CD8 T cells from both groups of mice were cytotoxic, though the human CD8 T cells from DRAGA mice were more efficient at killing target cells than their counterparts in A2 mice.

One mechanism by which cytotoxic CD8 T cells kill target cells is based on secretion of perforin and granzymes. Perforin creates pores in the membrane of target cells and allow the entry of granzymes that activate cell-death pathways^25^. We thus investigated the ability of human CD8 T cells from DRAGA and A2 mice to produce perforin and granzyme B by intracellular staining using specific antibodies. Figure 4c shows that the frequency of perforin^+^ CD8^+^ T cells in spleens of DRAGA and A2 mice was similar (p = 0.5), though the frequency of granzyme B^+^ CD8^+^ T cells in DRAGA mice was significantly higher than in the A2 mice (p = 0.02). In aggregate, these results indicated that as compared to expression of HLA-A2 molecules, co-expression of HLA-DR4 and HLA-A2 molecules significantly enhances the ability of human CD8 T cells to produce granzyme B and to kill target cells in a HLA-A2.1-restricted manner.

### Neither HLA-DR4 nor HLA-A2 expression in NRG mice affects the rate of reconstitution and numbers of human B cells

**Figure 5:**
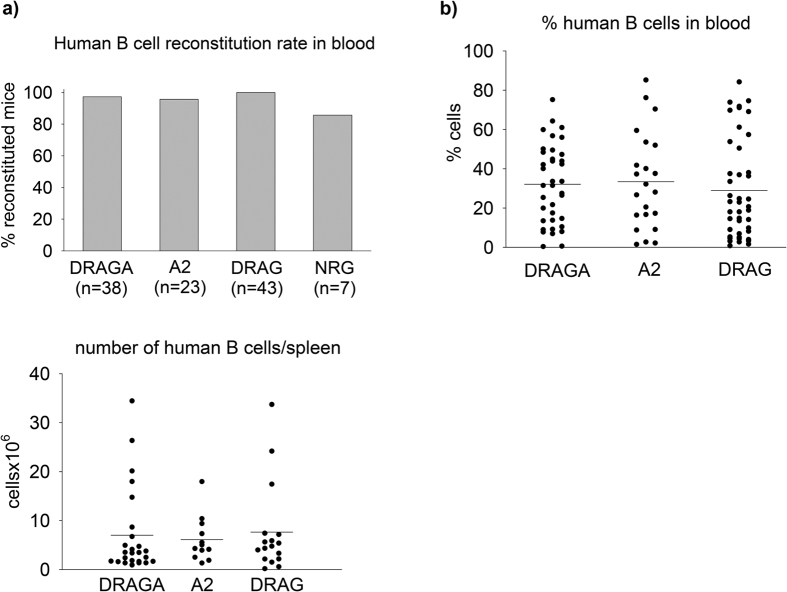
Neither HLA-DR4 nor HLA-A2 expression in NRG mice affects the rate of reconstitution and numbers of human B cells. Mice were examined for human B cell reconstitution by FACS using human CD19 Abs. Panel (**a)** shows the human B cell reconstitution rate (percentage of mice having human B cells in blood). The cut-off for positive human B cells was calculated as three times the standard deviation over the background levels of cells from naıve (non-HSC infused) DRAG mice that were stained with anti-human CD19 (0.11%). Z test indicated no significant differences for human B cell reconstitution rates among DRAGA (37 of 38), DRAG (43 of 43), A2 (22 of 23), and NRG (6 of 7) mice (p > 0.6). Panel (**b**) frequency of human B cells in blood of DRAGA, DRAG, and A2 mice. Panel (**c**) total numbers of human B cells in spleens of DRAGA, DRAG, and A2 mice. Data represent values in individual mice. Horizontal lines represent mean values. Student *t* test indicated no significant differences for numbers of human B cells among DRAGA, DRAG, and A2 mice (p > 0.5).

To address the role of HLA-A2 and HLA-DR4 molecules for reconstitution of human B cells, DRAGA mice (n = 38), DRAG mice (n = 43), A2 mice (n = 23), and non-Tg NRG mice (n = 7) were examined for human B cell reconstitution rates (percentage of mice able to develop human B cells in the blood). As illustrated in Fig. 5a, all groups of mice were highly proficient at reconstituting human B cells, since 37/38 DRAGA, 43/43 DRAG, 22/23 A2, and 6/7 NRG mice reconstituted human B cells in blood. Furthermore, the frequency of human B cells in the blood of DRAGA, DRAG, and A2 mice were similar (Fig. 5b). The total numbers of human B cells reconstituting the spleens were also comparable among the DRAGA, DRAG, and A2 mice (Fig. 5c). In aggregate, these results clearly indicated that neither HLA-A2 nor HLA-DR4 expression influences the ability of NRG mice to reconstitute human B cells.

### HLA-DR4, but not HLA-A2 expression in NRG mice supports B cell immunoglobulin class switching and secretion of IgG

**Figure 6:**
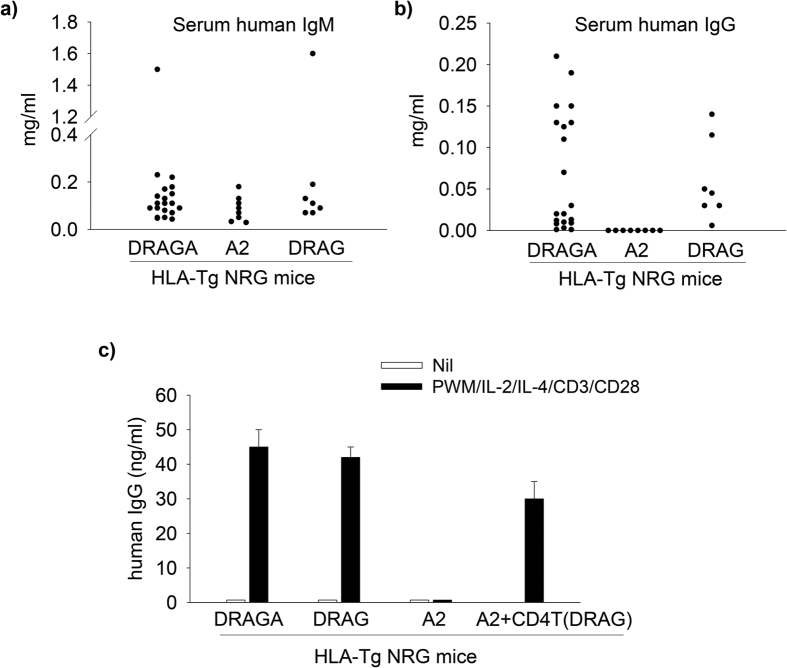
HLA-DR4, but not HLA-A2 expression on NRG mice supports B cell immunoglobulin class switching and secretion of IgG. Panel (**a,b**) At 16–18 weeks post-infusion of human HSCs, DRAGA, DRAG, and A2 mice were examined for serum levels of human IgM (**a**) and human IgG (**b**) by ELISA. Data show values in individual mice. Panel (**c**) splenic cells from DRAGA, DRAG, and A2 mice (n = 3 mice pooled) were stimulated *in vitro* with PWM plus human IL-2, IL-4 and human CD3/CD28 Abs for 16 days and the levels of human IgG secreted in cell culture supernatants were measured by ELISA. Splenic cells from A2 mice were also stimulated as above in the presence of purified CD4 T cells from DRAG mice. All mice used in this experiment were infused with HSC from the same donor. Data show mean ± SD.

We previously showed that the human B cells in NRG mice cannot undergo immunoglobulin class switching^12^. We also showed that the expression of HLA-DR4 molecules in NRG (DRAG) mice rescues the ability of human B cells to undergo immunoglobulin class switching and to reconstitute serum levels of human IgG^12^. To address the contribution of HLA-DR4 *vs* HLA-A2 molecules on human B cell immunoglobulin class switching, we measured the serum levels of human immunoglobulins (natural antibodies) in DRAGA and A2 mice by ELISA. As control we used DRAG mice. As illustrated in Fig. 6a, DRAGA and A2 mice reconstitute similar levels of serum human IgM as those in serum of DRAG mice. However, the DRAGA and DRAG mice reconstitute serum levels of human IgG while the A2 mice had no detectable serum IgG (Fig. 6b). The results demonstrated that the expression of HLA-DR4 molecules in NRG mice, but not HLA-A2 molecules, supports B cell immunoglobulin class switching.

### Human B cells in humanized NRG mice require functional human CD4 helper T cells to undergo immunoglobulin class switching

Cytokines secreted by CD4 helper T cells are required to support B cell immunoglobulin class switching from IgM to IgG^26^. The human CD4 T cells from DRAGA and DRAG mice were proficient at secreting cytokines, while the human CD4 T cells from A2 mice were not (Fig. 3). Thus we investigated whether the inability of human B cells from A2 mice to secrete IgG underlies a deficient human CD4 helper T cell function. For this, we conducted an *in vitro* assay in which splenic cells from DRAGA, DRAG, and A2 mice were stimulated with pokeweed mitogen (PWM) plus IL2/IL-4 and CD3/CD28 Abs and the levels of human IgG secreted in cell culture supernatants were measured by ELISA at day 16 post-stimulation. Of note, activation of B cells by PWM results in IgG secretion only in the presence of T cells, since PWM is a T cell-dependent B cell activator^27^. As illustrated in Fig. 6c, the PWM-stimulated splenic B cells from DRAGA and DRAG mice secreted IgG, while the splenic B cells from A2 mice failed to do so. However, when co-cultured with purified CD4 T cells from DRAG mice, the splenic B cells from A2 mice regained the ability to secrete IgG (Fig. 6c). The results clearly indicated that the inability of human B cells from A2 mice to secrete IgG underlies a deficient human CD4 helper T cell function. These results also explain the ability of human B cells from DRAGA and DRAG mice to secrete IgG *in vivo*, by virtue of reconstituting CD4 helper T cells able to support immunoglobulin class switching.

## Discussion

This study demonstrated that transgenic expression of HLA-DR4 molecules in NRG mice, but not HLA-A2 molecules, improves thymic engraftment with human pro-T cells, the rate of human T cell reconstitution (percentage of mice able to develop human T cells), the numbers of human CD4 T cells repopulating lymphoid organs, cytokine secretion by human CD4 and CD8 T cells, and B cell immunoglobulin class switching. Our study also showed that co-expression of HLA-DR4 and HLA-A2 molecules significantly increases the numbers and cytotoxic activity of HLA-A2-restricted human CD8 T cells as compared to expression of HLA-A2 molecules alone.

Thymopoiesis is a physiological process by which pro-T cells derived from the bone marrow undergo positive or negative selection upon TCR interaction with peptide-MHC complexes expressed by thymic epithelial cells^17^. Mature thymocytes then migrate from the thymus to repopulate secondary lymphoid organs such as blood, spleen, lymph nodes, and gut-associated lymphoid organs (GALT)^28^. As found in this study the expression of HLA-DR4 molecules in NRG mice, as compared to HLA-A2 molecules, enhanced the engraftment of human pro-T cells in the mouse thymus. This was evident since the thymus of DRAG and DRAGA mice contained significantly higher numbers of human thymocytes than the thymus of A2 mice. Interestingly, co-expression of HLA-A2 molecules and HLA-DR4 molecules in DRAGA mice did not increase the threshold of thymic engraftment as compared to the expression of HLA-DR4 molecules alone in DRAG mice. The high level of thymic engraftment in DRAG and DRAGA mice may thus account for the higher T cell reconstitution rates and the higher human CD4 T cell numbers in peripheral lymphoid organs as compared to the A2 mice.

The ability of HLA-class II (DR4) molecules to improve reconstitution of human CD4 T cells in the NRG mouse, as found in this study and in our previous study^12^, is in agreement with studies using NOG mice transgenic for HLA-DR4^13^ and NSG mice transgenic for HLA-DR1^14^. Interestingly, Suzuki *et al*.^13^ also demonstrated that infusion of HLA-DR-matched human HSC is critical for improving human T and B cell function, since humanized HLA-DR4 mice that were infused with HLA-DR-mismatched human HSC could not undergo immunoglobulin and to secrete human IgG.

The NRG, NOG, and NSG mice are on a NOD (non-obese diabetic) genetic background but they differ in the mutation needed to abolish the development of mouse T and B cells that is required to prevent rejection of human HSCs (RagKO mutation in the NRG mice and *scid* mutation in the NOG and NSG mice). Unlike the RagKO mutation, the *scid* mutation is leaky and over time leads to accumulation of mouse T and B cells^29^. These humanized mouse models also differ in the mutation needed to abolish mouse innate immunity, which is needed to enhance the engraftment of human HSCs. The NRG and NSG mice carry a full deletion of the IL2Rγc gene^3^, whereas the NOG mice have a partial deletion of the IL2R*g*c gene^30^, though both mutations have been proven to equally prevent the development of mouse innate cells (particularly mouse NK cells). Besides these genetic differences, there is consensus on the ability of HLA class II expressing NRG, NOG, and NSG mice (infused with HSC from cord blood) to improve reconstitution of human CD4 T cells^12^,^13^,^14^. In contrast, there is one study in NSG mice co-expressing HLA-DR1 and HLA-A2 transgenes (infused with HSC from fetal liver) which showed no improvement in the numbers of reconstituted human CD4 T cells as compared to non-Tg NSG mice^15^ (Table 1). Whether this discrepancy might relate to the use of HSC from fetal liver *vs* cord blood still remains to be investigated.

Notably, the rate of human T cell reconstitution (percentage of mice able to develop T cells) in the A2 mice was similar to that in non-Tg NRG mice. Also, the numbers of reconstituted human CD8 T cells was not improved by the expression of HLA-A2 molecules, since both DRAGA and A2 mice (expressing HLA-A2 molecules) reconstituted similar numbers of human CD8 T cells as the DRAG mice (which do not express HLA-A2 molecules). The inability of HLA-A2 molecules to improve human CD8 T reconstitution in NRG mice is in agreement with studies indicating that the same HLA-A2 transgene did not improve human CD8 T cell reconstitution in the NSG mice^6^,^7^,^8^.

The HLA-A2 transgene used to generate the A2 mice encodes for a hybrid human/mouse HLA-A2.1 molecule consisting of HLA-A2.1α1/α2 domains and mouse H-2D^b^ α3, transmembrane, and cytoplasmatic domains covalently linked to human β2-microglobulin (termed HHD)^16^. Two additional HLA class I transgenes have been tested in humanized mice, namely a fully human HLA-A2.1 molecule (termed Enge) in NSG mice^9^,^10^,^15^ and a fully human HLA-B51.1 molecule in NOK mice^11^ (Table 1). The NOK mice are NOD.*scid* mice having a Jak3KO mutation, which has been proven to reduce mouse innate immunity similarly to the IL2RγcKO mutation. These four studies also reported no improvement for human CD8 T cell reconstitution in the HLA-A2 (Enge) and HLA-B51 Tg mice as compared to non-Tg mice. Our study in NRG mice is thus in agreement with studies in NSG and NOK mice indicating a minimal effect of transgenic HLA class I expression for human CD8 T cell reconstitution.

The DRAG, DRAGA, and A2 mice express mouse MHC class II (IA^g7^) molecules and mouse MHC class I (H-2 K^d^, H-2D^b^) molecules. The expression of mouse IA^g7^ (class II) as compared to HLA-DR4 contributed minimally to the reconstitution and function of human CD4 T cells, since DRAGA and DRAG mice (expressing HLA-DR4 and IA^g7^) reconstituted significantly higher numbers of human CD4 T cells than A2 mice (expressing only mouse IA^g7^). Importantly, expression of HLA-DR4 molecules in DRAGA and DRAG mice resulted in the development of human CD4 T and CD8 T cells that were proficient at secreting cytokines, while the expression of IA^g7^ molecules alone in the A2 mice did not suffice to do so. Thus, our results clearly indicated that expression of HLA-DR4 molecules in NRG mice is critical for supporting reconstitution of human CD4 T and CD8 T cells able to secrete cytokines.

On the other hand, co-expression of HLA-A2 and mouse MHC class I molecules did not enhance the reconstitution of human CD8 T cells, since DRAG mice (which do not express HLA-A2) reconstituted similar numbers of human CD8 T cells as the DRAGA and A2 mice (which express HLA-A2). However, the human CD8 T cells from DRAGA and A2 mice were restricted by HLA-A2 molecules, which indicated that expression of HLA-A2 in thymus had a role in education of human CD8 T cells. This result is in agreement with other studies using HLA class I-expressing NSG and NOK mice, which proved to elicit HLA class I-restricted human CD8 T cells upon vaccination or infection^6^,^7^,^8^,^9^,^10^,^11^,^15^. Importantly, we found that co-expression of HLA-DR4 and HLA-A2 molecules in DRAGA mice significantly increased secretion of granzyme B and the cytotoxic activity of the human CD8 T cells as compared to the A2 mice. Several cytokines, including IL-2, IL-10, and IFNγ secreted by CD4 T cells induce granzyme B expression on CD8 T cells^31^. The fact that human CD4 T cells from DRAGA mice were proficient at secreting human cytokines may thus explain the higher cytotoxicity of their human CD8 T cells as compared to those in the A2 mice.

Reconstitution of human B cells in humanized mice is not dependent on the expression of HLA class I or HLA class II molecules as demonstrated in our prior study using NRG mice^12^ and in other studies using NSG and NOK mice^6^,^7^,^8^,^9^,^10^,^11^,^12^,^13^,^14^,^15^, since all these mouse strains were proficient at reconstituting human B cells. However the human B cells from humanized NRG, NSG, and NOK mice are arrested in development and cannot undergo immunoglobulin class switching^4^,^5^,^12^,^32^. Importantly, we and others have shown that expression of HLA class II molecules but not HLA class I molecules, rescues the ability of human B cells to undergo immunoglobulin class switching and to secrete IgG^12^,^13^,^14^ (Table 1). Cytokines secreted by CD4 helper T cells such as IL-4, IFNγ, IL-5, and TGFβ regulate immunoglobulin class switching by modulating transcription of germline Ig-CH (constant) genes on B cells^26^. Although cytokines alone do not induce immunoglobulin class switching, the induction of germline transcription by cytokines is a critical checkpoint that precedes immunoglobulin class switching after B cell activation^26^. The ability of DRAG and DRAGA mice to reconstitute human CD4 T cells that secrete human cytokines accounts for the ability of their human B cells to undergo immunoglobulin class switching and to secrete IgG. This was evident since the human B cells from A2 mice, which cannot undergo class switching, regained the ability to secrete IgG upon co-culture with purified human CD4 T cells from DRAG mice.

In aggregate our results indicated a multifactorial effect of the HLA-DR4 transgene on thymic engraftment and development of functional human CD4 T cells, which support immunoglobulin class switching and the cytotoxic activity of antigen-specific human CD8 T cells.

## Methods

### Ethics Statement

All animal procedures reported herein were conducted under IACUC protocols approved by WRAIR/NMRC and USUHS in compliance with the Animal Welfare Act and in accordance with the principles set forth in the “Guide for the Care and Use of Laboratory Animals,” Institute of Laboratory Animals Resources, National Research Council, National Academy Press, 1996.

### Generation of HLA-transgenic NRG mice

The DRAG mice (NOD.HLA-DR4.RagKO.IL2RgcKO) express HLA-DR4 molecules in NRG background, and they have been previously described^12^. The DRAG mice were crossed with HLA-A2.1.NOD mice (stock #006611, The Jackson Laboratory) to generate F1 mice that were intercrossed to generate F2 mice. The F2 progeny was screened for expression of HLA-DR4 and HLA-A2 molecules by FACS using peripheral white blood cells stained with HLA-DR and A2 antibodies (clones #Tu39 and #BB7.2, BD Biosciences, San Jose, CA). Rag1KO litters were identified by the absence of mouse T cells upon staining of peripheral white blood cells with anti-mouse CD3 (clone #145-2C11, BD Biosciences). The IL2RγcKO mutation was screened by PCR as previously described^12^. Mice were bred at the Veterinary Program Service at NMRC/WRAIR.

### Infusion of mice with HSC

Umbilical cord bloods positive for HLA-DR4 (B1*0401) and HLA-A2.1 were obtained from the NY Blood Center, Long island City, NY. Procedures for isolation of human CD34^+^ hematopoietic stem cells have been previously described^12^. Human HSC cell viability was >95% as determined by Trypan blue exclusion. At the age of 4–6 weeks, groups of DRAGA, DRAG, and A2 mice were injected intravenously with CD34^+^ HSC (10^5^ per mouse) from the same donors (Supplementary Table S1). Mice were used at 16–18 weeks after the infusion of HSCs.

### FACS analysis

Blood drawn for the tail vein (50 μl) was collected with heparin-coated capillary tubes (Fisher Scientific, Pittsburgh, PA). Erythrocytes were lysed using ACK lysis buffer (Invitrogen, San Diego, CA) and white blood cells were analyzed by FACS using specific antibodies^12^. Splenic and thymic cells were isolated as described^1^,^12^. Cell populations were quantified by FACS on mononuclear FSC/SSC gating using anti-human CD45 (clone #2D1), CD3 (#HIT3a), CD4 (#SK3), CD8 (#RPA-T8), and CD19 (#H1B19), HLA-DR (#tu39), HLA-A2 (#BB7.2), and intracellularly stained with granzyme B (#GB11) (BD Biosciences, San Diego, CA), perforin (#B-D48, BioLegend, San Diego, CA) or FOXP3 (#PCH101, eBiosiences, San Diego, CA) Abs. HLA-A2/GIL dextramers were obtained from Immudex (Copenhagen, Denmark) and GIL-specific CD8 T cells were enumerated by FACS following the manufacturer’s instructions.

### T cell responses

Splenic cells (5 × 10^5^) were stimulated for 36 h with 20 ng/ml PMA plus 1 μM, ionomycin (Sigma), or stimulated for 72 h with anti-human CD3/CD28 coated beads (Dynabeads human T cell activator CD3/CD28, Invitrogen), or left unstimulated. Cytokines in supernatants were measured by Luminex (Invitrogen).

### Cell depletion and isolation

Spleen cells adjusted to 5 × 10^6^ cells/ml were depleted of CD4 T cells or CD8 T cells with antibody-coated magnetic beads (Dynabeads, Invitrogen). The efficiency of cell depletion was more than 95% as measured by FACS using CD3, CD4, and CD8 Abs. Equal volumes (0.1 ml) of cell depleted cell suspensions or corresponding volumes of positively isolated cells on the magnetic beads were stimulated with PMA/ionomycin for 36 h and cytokine secretion in cell culture supernatants were measured by Luminex (Invitrogen).

### Immunoglobulins in plasma

Peripheral blood was collected in heparin-coated capillaries and plasma was obtained by removal of cells by centrifugation. Human immunoglobulins were measured by ELISA Quantitation Sets (Bethyl Labs, Montgomery, Texas).

### Immunization

The synthetic GIL (GILGFVFTL) peptide corresponds to the influenza A virus matrix protein, Mp58–66 and was obtained from Mimotopes Inc. The GIL peptide was encapsulated in liposomes containing neutral and anionic saturated phospholipids, cholesterol, and monophosphoryl lipid A (MPLA) as described^33^. The liposomes were obtained from the Laboratory of Adjuvant and Antigen Research, US Military HIV Research Program, Walter Reed Army Institute of Research. Mice were immunized intramuscularly twice at two weeks apart with 200 μg of GIL peptide encapsulated in 100 μl of liposomes. Mice were euthanized 10 days after the last immunization.

### CTL assay

Splenic cells from GIL-immunized mice were stimulated *in vitr*o with GIL peptide (2 μg/ml) plus IL-2 (20 U/ml, Life Technologies) for 7 days. T2 cells (ATCC) were used as target cells and they were pulsed with GIL peptide (40 μg/ml) and ^51^Cr (100 μCi/ml/1 × 10^6^) for 4 h, washed three times and incubated at effector:target ratios of 20:1 7:1, and 3:1 in 96-well U bottom plates for 4 h. The percentage of specific lysis was calculated as 100x[(experimental-spontaneous)/(maximum-spontaneous)].

### *In vitro* IgG secretion

Splenic cells (2.4 × 10^5^) were stimulated with Pokeweed mitogen (PWM, 40 μg/ml, Sigma, St Louis, MO), human IL-2 (15 U/ml), IL-4 (100 U/ml) (Life Technologies), and human CD3 and CD28 Abs (1 μg/ml each, BD Biosciences). At day 7 of culture, half of the medium was replaced by fresh medium. Immunoglobulin secretion in supernatants at day 16 of culture was measured by an enzyme-linked immunosorbent assay (ELISA) (human IgG ELISA quantitation set; Bethyl Laboratories,). For co-cultures, negatively isolated human CD4 T cells (7.5 × 10^4^) from DRAG mice (CD4 dynabeads, Invitrogen) were added to the splenic cells of A2 mice and stimulated as above.

### Statistical analysis

Data were analyzed using unpaired (2-tailed) Student *t* test, or Z-test 2-tailed at significant level of 0.05.

## Additional Information

**How to cite this article**: Majji, S. *et al*. Differential effect of HLA class-I *versus* class-II transgenes on human T and B cell reconstitution and function in NRG mice. *Sci. Rep.* **6**, 28093; doi: 10.1038/srep28093 (2016).

## Supplementary Material

Supplementary Information

## Figures and Tables

**Table 1:** Comparison of human immune cell function in HLA-Tg humanized mice vs non-Tg mice.

Mouse strain	HLA transgene	Age	Radiation dose (Rads)	Route	Source of HSC	#HSC injected	Improvement of				Ref.#
							T cell reconstitution	Polyclonal T cell cytokine response (stimulator)	HLA-restriction	Ig class switching	
NSG	A*2.01-Tg (HHD)	newborn	150	i.v.	CD34 cord blood	5–30 × 10^3^	No	No (PMA/Iono)	Yes	NR	6
		adult	240		T-cell depleted cord blood	3 × 10^4^	NR	NR	Yes	No	7
			300		CD34 bone marrow	4.6 × 10^5^	No	NR	Yes	NR	8
	A*2.01-Tg (Enge)	newborn	200	i.h.	CD34 cord blood	1 × 10^5^	No#	NR	NR	NR	9
			100		CD34 fetal liver	1–3 × 10^5^	No	No (SEB)	Yes	No	10
NOK^&^	B*51.1-Tg	newborn	NR		CD34 cord blood	5–10 × 10^4^	No^&^	NR	NR	NR	11
NRG	DR*0401-Tg	adult	350	i.v.		1 × 10^5^	Yes	Yes (PMA/Iono) Yes CD3/CD28)	NR	Yes	12
NOG	DR*0405-Tg Ab^0^		120			1 × 10^5^	Yes	Yes (PMA/Iono)	NR	Yes	13
NSG	DR*0101-Tg Ab^0^	newborn	150	i.h.		3–5 × 10^4^	Yes	Yes (PMA/Iono)	NR	Yes	14
	A*2.01-Tg (Enge) DR*0101-Tg		100		CD34 fetal liver	1.5–2 × 10^5^	No	Yes (PMA/Iono)	Yes	Very low	15

NSG, NOD.*scid*.IL2R*g*cKO (complete IL2Rγc mutation); NOG, NOD.*scid*.IL2R*g*cKO (truncated IL2Rγc mutation). NRG, NOD.Rag1KO.IL2RγcKO (complete IL2Rγc mutation); NOK, NOD.*scid*.Jak3KO; HHD, hybrid human/mouse HLA-A2.1/H2D^b^ covalently linked to human β2 m; Enge, human HLA-A2.1; Ab^0^, mouse MHC-II KO; i.v., intravenous; i.h., intrahepatic; NR, not reported; (^#^) this study showed lower human T cell reconstitution rate in HLA-A2-Tg NSG mice as compared to NSG mice; (^&^) No HLA genotyping was performed on cord bloods used for this study.
