# Supplementary material for: Differential effect of HLA class-I versus class-II transgenes on human T and B cell reconstitution and function in NRG mice
Source: Sci Rep. 2016 Jun 21;6:28093. doi: 10.1038/srep28093 (PMC4914985; doi:10.1038/srep28093)
Supplement: Supplementary Information [file srep28093-s1.pdf]

Title: Differential effect of HLA class-I *versus* class-II transgenes on human T and B cell reconstitution and function in NRG mice

Authors: Sai Majji, Wathsala Wijayalath, Soumya Shashikumar, Luis Pow-Sang, Eileen Villasante, Teodor D. Brumeanu and Sofia Casares

Supplementary Table S1.HLA genotype of cord bloods used for reconstitution of mice

| Cord blood* | HLA-A | HLA-DRB1 | Number of infused mice | | | |
|---|---|---|---|---|---|---|
| | | | DRAGA | A2 | DRAG | NRG |
| Donor #1 | 02:01<br>33:01 | 04:0101<br>15:01 | 11 | 6 | 9 | 2 |
| Donor #2 | 02:01<br>01:01 | 04:0101<br>04:04 | 11 | 3 | 13 | 0 |
| Donor #3 | 02:01<br>29:01 | 04:0101<br>03:01 | 9 | 9 | 13 | 2 |
| Donor #4 | 02:01<br>29:01 | 04:0101<br>13:0501 | 8 | 5 | 0 | 0 |
| Donor #5 | 02:01<br>03:01 | 04:0101<br>08:01 | 0 | 2 | 0 | 0 |
| Donor #6 | 02:01<br>01:01 | 04:0101<br>11:0102 | 4 | 5 | 0 | 0 |
| Donor #7 | 02:01<br>01:03 | 04:0101<br>15:0101 | 4 | 0 | 8 | 3 |

DRAGA, NOD.HLA-A2.HLA-DR4.RagKO.IL2RγcKO; DRAG, NOD.HLA-DR4.RagKO.IL2RγcKO; A2, NOD.HLA-A2.RagKO.IL2RγcKO, NRG, NOD.RagKO.IL2RγcKO.
